# Ionophores for Reference Electrodes Based on Organic Electrolytes

**DOI:** 10.1021/acs.analchem.5c04958

**Published:** 2025-11-07

**Authors:** Nikolai Yu. Tiuftiakov, Eric Bakker

**Affiliations:** Department of Inorganic and Analytical Chemistry, 27212 University of Geneva, Quai Ernest-Ansermet 30, CH-1211 Geneva, Switzerland

## Abstract

Reference electrodes (REs) based on moderately lipophilic electrolytes form an attractive principle for miniaturized, maintenance-free sensing systems. Unfortunately, their broader adoption has been hampered by shortcomings related to limited lifetime and sample contamination from electrolyte leaching as well as poor compatibility with complex sample matrices. While exploring highly lipophilic electrolytes offers a potential solution, the performance of the corresponding REs is compromised by a mismatched stoichiometric composition of the used salt. To address this, we describe here a novel potential stabilization mechanism that capitalizes on the presence of an ion mismatch in the reference electrolyte by incorporating an ionophore into the membrane. In contrast to conventional applications, combining ionophores with mismatched organic electrolytes generates a sample-independent interfacial potential through a combination of complexation and ion-exchange processes at the membrane/sample interface. The mechanism is investigated in more detail through theoretical simulations, and the approach is validated experimentally using commercially available lipophilic salts and potassium-selective valino­mycin as the ionophore. While the resulting REs require further optimization for practical use, they already exhibit notable stability in KCl solutions, with response slopes as low as 0.4 ± 0.2 mV and only 2.7 mV variation across repeat calibrations over a wide concentration range.

## Graphical abstract

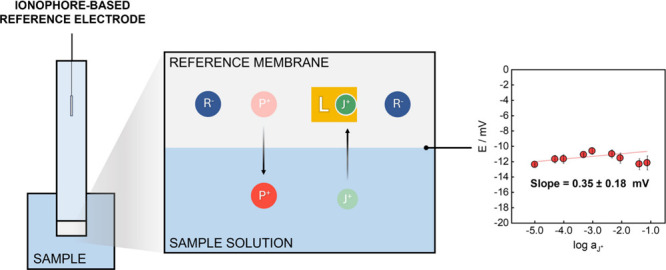

Modern electrochemical sensor development today focuses on compact, often wearable devices and platforms for point-of-care medical testing and *in situ* environmental analysis. Potentiometric, amperometric and other readout principles have been adapted for the quantification of various analytes in biological fluids and natural water samples, bringing forth advancements in electrochemical recognition mechanisms.^1,2^ However, this progress has also highlighted the need for new robust and versatile reference electrode designs. Although liquid contact Ag/AgCl-based electrodes remain widely used owing to their availability and well-documented stability, they are not well-suited for many applications due to limitations pertaining to miniaturization, inner solution management and mechanical stability under high-pressure conditions.^2^

These shortcomings have contributed to the increasing interest in liquid-junction-free reference electrodes (REs) based on organic electrolytes. In such REs, conventional aqueous bridge electrolytes are substituted with water-immiscible salt bridges, most often in the form of polymeric membranes. An organic electrolyte contained in the hydrophobic phase establishes an equilibrium distribution across the bridge/sample phase interface and thus governs the corresponding potential, rendering it independent of solution composition.^3–5^ The elimination of the liquid junction and the versatility of the hydrophobic bridge significantly simplify RE design and improve scalability, as demonstrated in a multitude of contributions leveraging this general principle to develop compact REs suitable for different analytical tasks.^6–9^

Among the tested liquid junction-free RE components, ionic liquids and solid organic salts of moderate lipophilicity have found the most use. Their relatively high aqueous solubility (typically around 1 mM) ensures sufficient salt transfer across the phase boundary, which in turn positively impacts RE potential stability and reproducibility.^4,10,11^ Unfortunately, these benefits come with trade-offs. The continuous leaching of the electrolyte into the aqueous sample limits RE lifetime and may interfere with the response of polymeric membrane ion-selective electrodes (ISEs) used within the same device.^12,13^ Although approaches mitigating this effect have been reported, they typically come at the expense of simplicity of the RE design and fabrication.^14^ Furthermore, ionic lipophilic interferences in the sample may also affect the RE potential via ion-exchange, which limits the applicability of the corresponding electrodes to the analysis of samples with complex matrices.^12^

Increasing the lipophilicity of the organic electrolyte could be doubly advantageous: a more lipophilic salt is better retained within the reference membrane, and improved ion lipophilicity reduces the ion-exchange competition with ionic solution species. Unfortunately, highly lipophilic electrolytes have not yet been successfully applied. A limited number of studies report adequate potential stability in NaCl and KCl solutions, yet poor performance in the presence of lipophilic interferences, contrary to expectations.^4,15^

In recent work, we established a direct link between this unexpectedly poor performance and the compositional purity of the organic electrolyte.^16^ The observed RE response appears to be a consequence of the slight mismatch of the lipophilic ions comprising the salt, likely originating from the metathesis synthesis process. Such a compositional mismatch is less consequential with moderately lipophilic electrolytes because of their effective partitioning into the aqueous phase. In contrast, an excess of a highly lipophilic ion remains confined to the hydrophobic phase and may act as an ion-exchanger. Notably, critical changes to RE stability behavior may be observed already at mismatches as low as 0.1% of the total lipophilic electrolyte loading,^16^ imposing extremely stringent—and often impractical—requirements on the salt purification procedure.

In this work, we propose an alternative strategy that suppresses the negative influence of lipophilic electrolyte mismatch in RE design. The approach is based on the incorporation of an ionophore into the reference membrane alongside a mismatched organic salt. This effectively retains the mismatched electrolyte in the membrane and mitigates its influence on the phase boundary potential. The interfacial potential becomes stable across a wide range of concentrations of the ion being complexed by the ionophore.

The general principle is illustrated in Figures 1 and 2 for a lipophilic electrolyte P^+^R^–^ characterized by a 10% excess of the anionic component. When a reference membrane containing a mismatched electrolyte only (no added ionophore) is brought into contact with an aqueous sample, the available anionic sites—much like in classical ion-exchanger based ion-selective membranes—are occupied by the dominant cation of the sample matrix (J^+^) (see Figure 1a). As a result, the concentration of J^+^ within the membrane becomes fixed, giving rise to a Nernstian response with respect to its activity in the sample. This is illustrated in the simulated potential response shown in Figure 1b (for simulation details, see the Supporting Information, eqs S1–12). The predicted electrode response features a potential plateau at low J^+^ concentrations, from which a linear Nernstian segment gradually emerges as the concentration increases.

**Figure 1:**
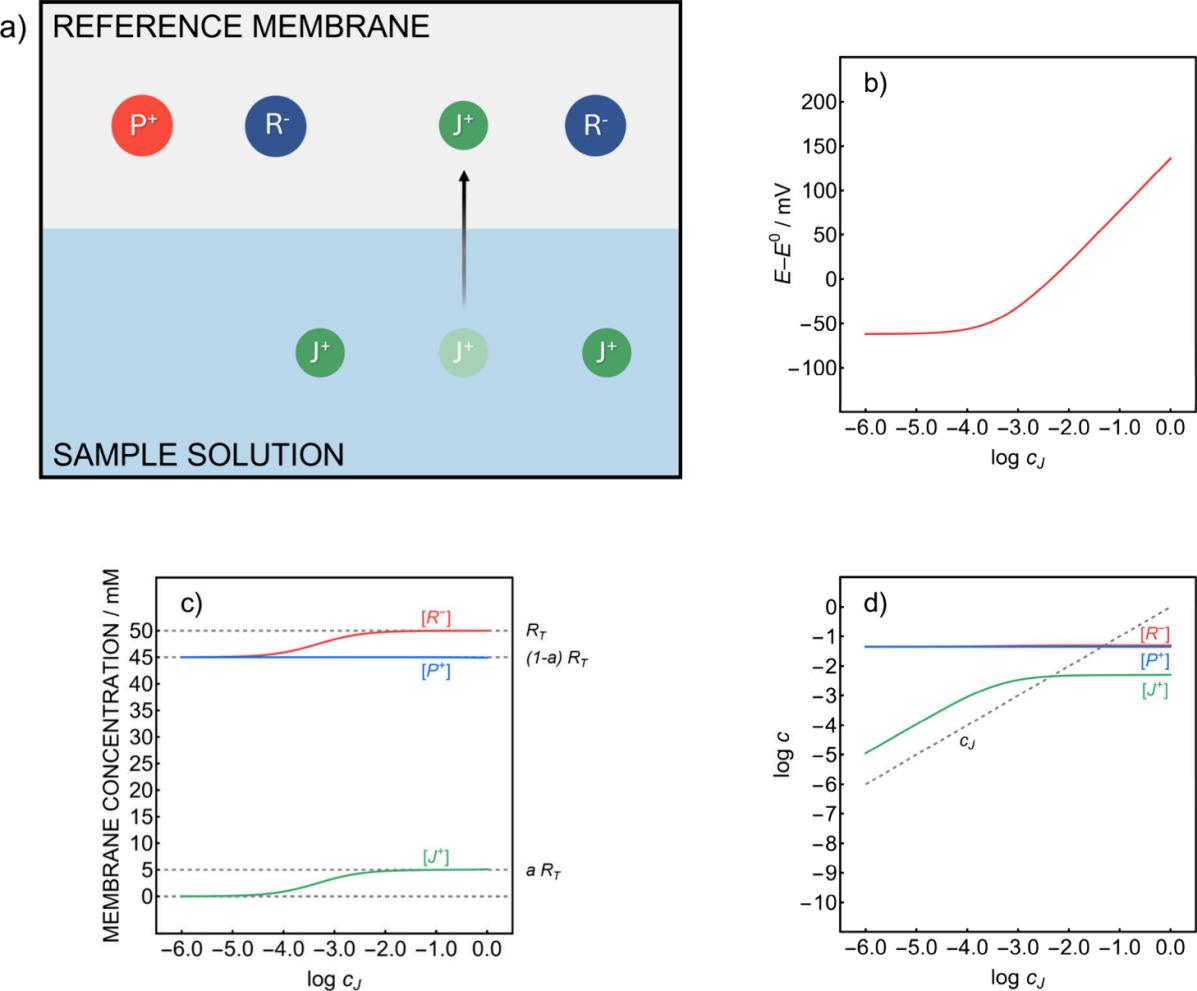
(a) Schematic interface equilibria for a reference membrane containing a slightly mismatched highly lipophilic electrolyte P^+^R^–^, where J^+^ represents the dominant sample cation. Simulated RE potential response curve (b) and linear (c) and logarithmic (d) membrane concentration profiles for the corresponding membrane composition as a function of the sample concentration of J^+^ (for simulation parameters, see the Supporting Information). *R*_*T*_ represents the total concentration of the lipophilic electrolyte in the membrane and *a* is the fraction corresponding to the mismatched anion. Square brackets denote membrane concentrations of the corresponding species, *c*_*J*_ is the J^+^ concentration in the sample.

**Figure 2:**
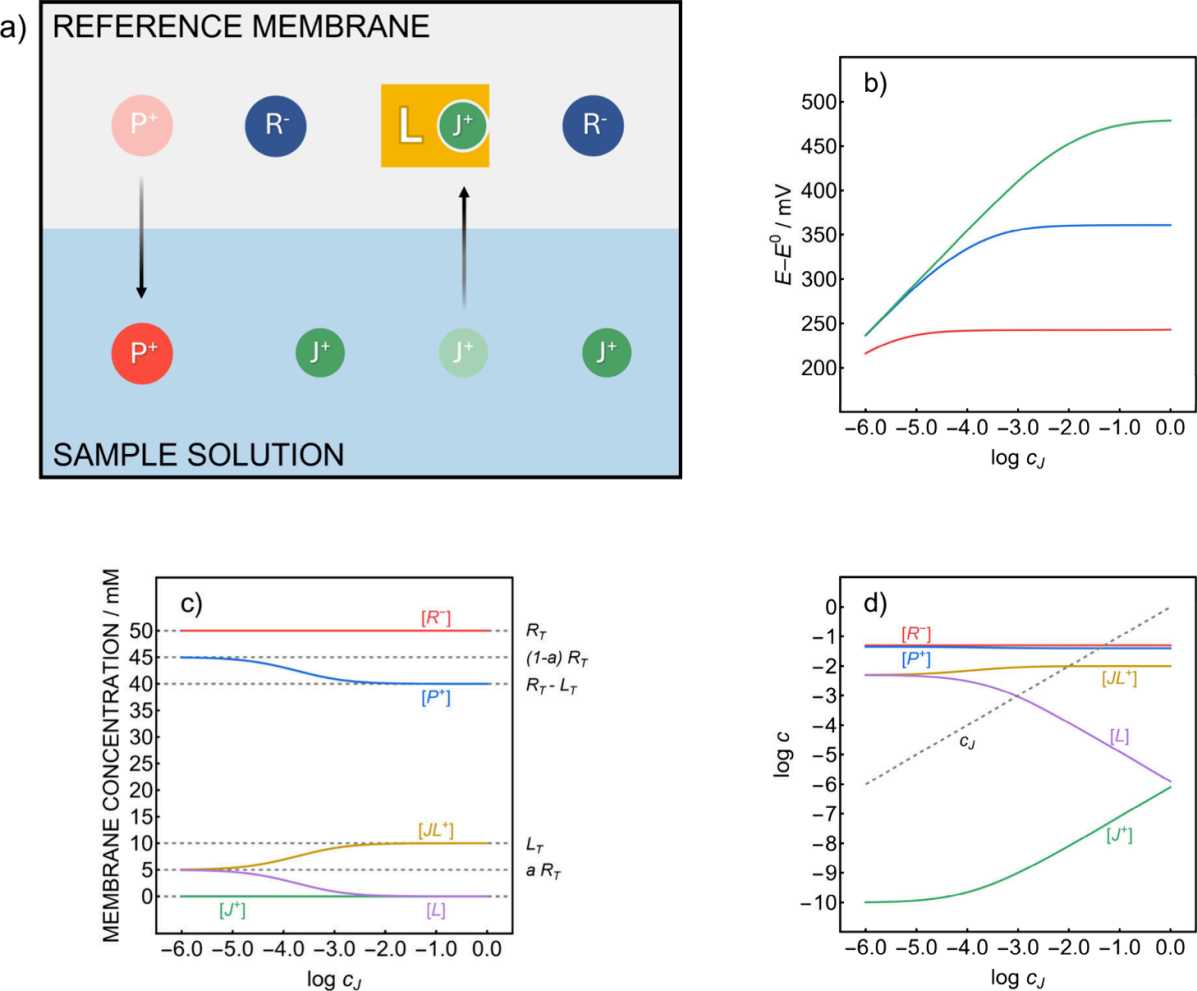
(a) Schematic interface equilibria for a reference membrane containing a slightly mismatched highly lipophilic electrolyte P^+^R^–^ and an ionophore L selective to a cation J^+^. Simulated RE potential response curves (b) and linear (c) and logarithmic (d) membrane concentration profiles for the corresponding membrane composition as a function of the sample concentration of J^+^. The concentration profiles in (c) and (d) were simulated for the composition corresponding to the blue trace in (b) (for simulation parameters, see the Supporting Information). *R*_*T*_ and *L*_*T*_ represent the total membrane concentrations of the lipophilic electrolyte and the ionophore, respectively, and *a* is the fraction corresponding to the mismatched anion. Square brackets denote membrane concentrations of the corresponding species, and *c*_*J*_ is the J^+^ concentration in the sample.

The origin of the stable potential at low sample concentrations can be understood by the simulated membrane concentration profiles in Figure 1c. The plateau region coincides with the range in which J^+^R^–^ partitioning into the sample becomes dominant, resulting in the breakdown of permselectivity.

The logarithmic membrane concentration profiles (Figure 1d), in turn, show that J^+^R^–^ partitioning establishes a concentration range in which the membrane concentration of J^+^ becomes proportional to its aqueous counterpart. This is the reason for constant potential response according to the phase boundary potential (see Supporting Information, eq S7). However, with highly lipophilic salts, J^+^R^–^ is unlikely to dominate the vicinity of the membrane owing to its low concentration and is therefore of limited practical value for the fabrication of reference electrodes.

Beyond the experimentally relevant range of J^+^ concentrations, a brief transition at extremely high experimentally unattainable concentrations of J^+^ is simulated followed by a return to Nernstian behavior (Figure S1a). As shown in Figure S1b,c, this transition indicates the point at which J^+^ displaces the lipophilic cation P^+^ from the organic phase until all anionic sites in the membrane are occupied by J^+^ only. This predicted behavior is analogous to the phenomena described previously for REs based on moderately lipophilic ionic liquids,^12^ although here it is expected at much higher sample concentrations due to substantially higher salt lipophilicity.

The original goal of the approach introduced here was to suppress the partitioning of the mismatched salt J^+^R^–^ into the aqueous phase so that instead the partitioning of the organic electrolyte P^+^R^–^ could become potential-determining. Partitioning of J^+^R^–^ not only depends on the lipophilicity of the two ions but also on their so-called free concentration.^17^ Accordingly, the concentration of free J^+^ in the membrane can be reduced by orders of magnitude by incorporating an ionophore L. The dominant form of J^+^ is now the complex JL^+^ and its dissociation to J^+^ and L is given by the complex formation constant. The partitioning of mismatched salt J^+^R^–^ may therefore be sufficiently suppressed, as intended.

At first glance, however, the incorporation of an ionophore L selective to J^+^ into the membrane alongside the mismatched lipophilic salt appears to have little impact on the behavior of the corresponding RE. Although the transfer of J^+^ into the membrane is facilitated by the new complexation equilibrium, its concentration remains fixed by the anionic sites provided by the excess of R^–^. The same quantity of J^+^ is extracted into the membrane, now simply existing in the form of the JL^+^ complex with a small fraction of the free ion still present in the membrane (Figure 2a). One may expect a Nernstian response to the sample cation, independent of the properties of the used electrolyte, as with a routine potentiometric probe. This is clearly not desired. As shown in Figure 2b, however, a sample-independent potential plateau that depends on the lipophilicity of the organic electrolyte (increasing from red to green trace) is indeed predicted, which is perhaps not immediately intuitive.

For the principle to work, the ionophore should not be in molar excess to the lipophilic electrolyte P^+^R^–^ but only in moderate excess to the mismatch, see Figure 2c. In this case only, an increasing sample concentration of J^+^ will start to displace P^+^ from the membrane until most L is used to form the complex JL^+^. Normally such ion-exchange occurs at much higher concentrations (see Figure S1) but here the process is promoted by the complexation of the exchanged sample cation by the ionophore and continues until the latter is nearly fully consumed.

This is now different from the composition of a potentiometric sensing membrane since the concentration of free L begins to change together with that of J^+^ in the sample. As shown in Figure 2d, [*L*] decreases linearly with increasing sample *c*_*J*_, resulting in a proportionality of the J^+^ concentration between the membrane and the sample phase. Because of this, the phase boundary potential becomes sample independent, thereby forming a promising basis for a reference electrode containing a lipophilic organic electrolyte.

Simulated curves extended across a wider concentration range (Figure S2a-c) reveal that the upper boundary of the potential plateau remains limited by the concentration at which J^+^ displaces the remaining P^+^ from the organic phase. It is the same transition previously observed for ionophore-free membranes, and the similarity highlights the crucial role of the added ionophore: it effectively broadens the transition domain between a P^+^-dominated and a J^+^-dominated membrane state and establishes a wider concentration range where the interfacial potential becomes independent of sample composition.

The calculations also show that the sample concentrations of P^+^ and R^–^ remain constant in the J^+^ range corresponding to the sample-independent, constant potential RE response (Figure S 2d). This further confirms reference electrode behavior, as the concentrations of the respective species in the membrane are also maintained constant over the same region (Figures 2c-d and S2c). Based on this observation, a simplified analytical expression was derived describing the transition point from Nernstian to reference electrode response, following the framework previously developed to treat ISE detection limits and RE stability breakdown (for the full derivation, see the Supporting Information, eqs S13–S20):

1 $$c_{J^{+}}\left(transition\right)=\frac{aR_{T}}{K_{J/P}\;\beta q\left(R_{T}-L_{T}\right)}$$

where *R*_*T*_ and *L*_*T*_ are the total membrane concentrations of the lipophilic electrolyte and the ionophore respectively, *a* is the organic salt mismatch parameter, *K*_*J*/*P*_ is the equilibrium constant describing ion-exchange between P^+^ and J^+^, β is the stability constant of the complex JL^+^, *q* is the volume ration of the aqueous and the polymeric phase.

For the simulation parameters used, eq 1 predicts logarithmic transition concentrations of −5.9 (corresponding to the red trace in Figure 2b), −3.9 (blue trace in Figure 2b) and −1.9 (green trace in Figure 2b). These estimates are in full agreement with the corresponding values obtained directly from the simulated curves (Table S1). Furthermore, eq 1 explicitly indicates how different system parameters influence the position of the potential plateau. Smaller mismatches allow for lower ionophore loadings, both of which decrease the transition point concentration. Similarly, stronger complexation of the solution ion by the ionophore shifts the plateau toward lower concentrations. The effect of the electrolyte lipophilicity is more complex: although less lipophilic electrolytes may yield more practical potential stability ranges, this comes at the expense of limited electrode lifetime, which is not considered in the present model.

To experimentally validate the suggested potential stabilization mechanism, we selected three lipophilic electrolytes composed of different tetraalkyl­ammonium cations: tetrabutyl­ammonium (TBA^+^), tetrapentyl­ammonium (TPA^+^) and tetrahexyl­ammonium (THA^+^), each paired with the tetrakis­(4-chlorophenyl)­borate anion (T*p*ClPB^–^) (Figure 3). The partitioning and ion-exchange properties of the selected ions reflected the corresponding parameters used in the simulations, whereas varying the alkyl side chain length of the cation allows for straightforward tuning of the overall lipophilicity of the organic salt. During the initial performance screening, membranes were prepared by directly mixing the commercial salts of the corresponding ions into the membrane cocktail in a ratio corresponding to 50 mmol kg^–1^ of total salt with a 10% molar excess of T*p*ClPB^–^. No further purification was performed. The KCl response of the resulting membranes was then evaluated first as is and then with additional doping with 10 mmol kg^–1^ of K^+^-selective valino­mycin (Figure 4).

**Figure 3:**
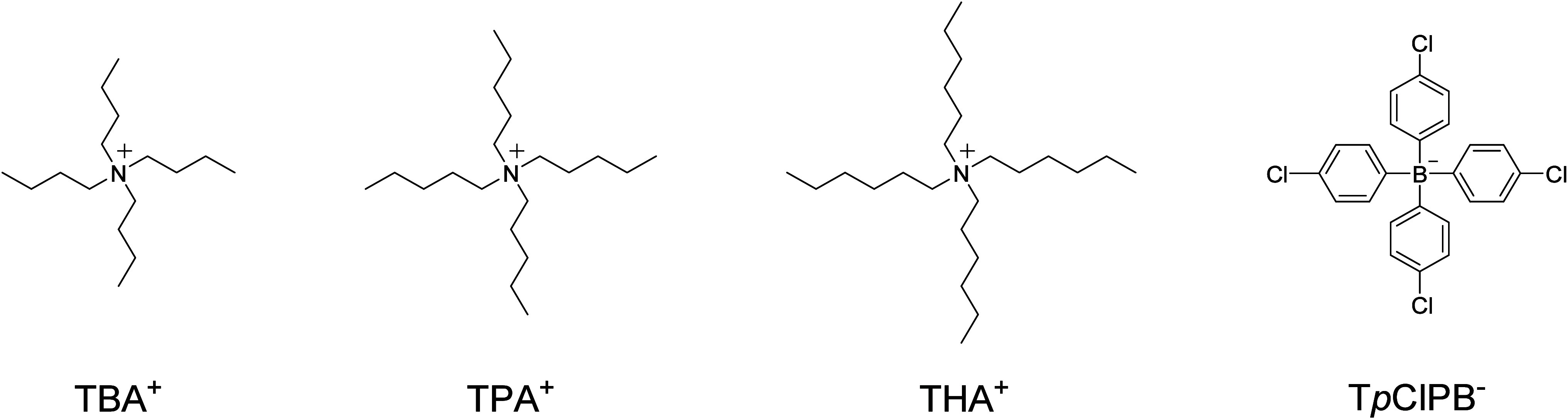
Structures of the ions composing lipophilic salts used in this study.

**Figure 4:**
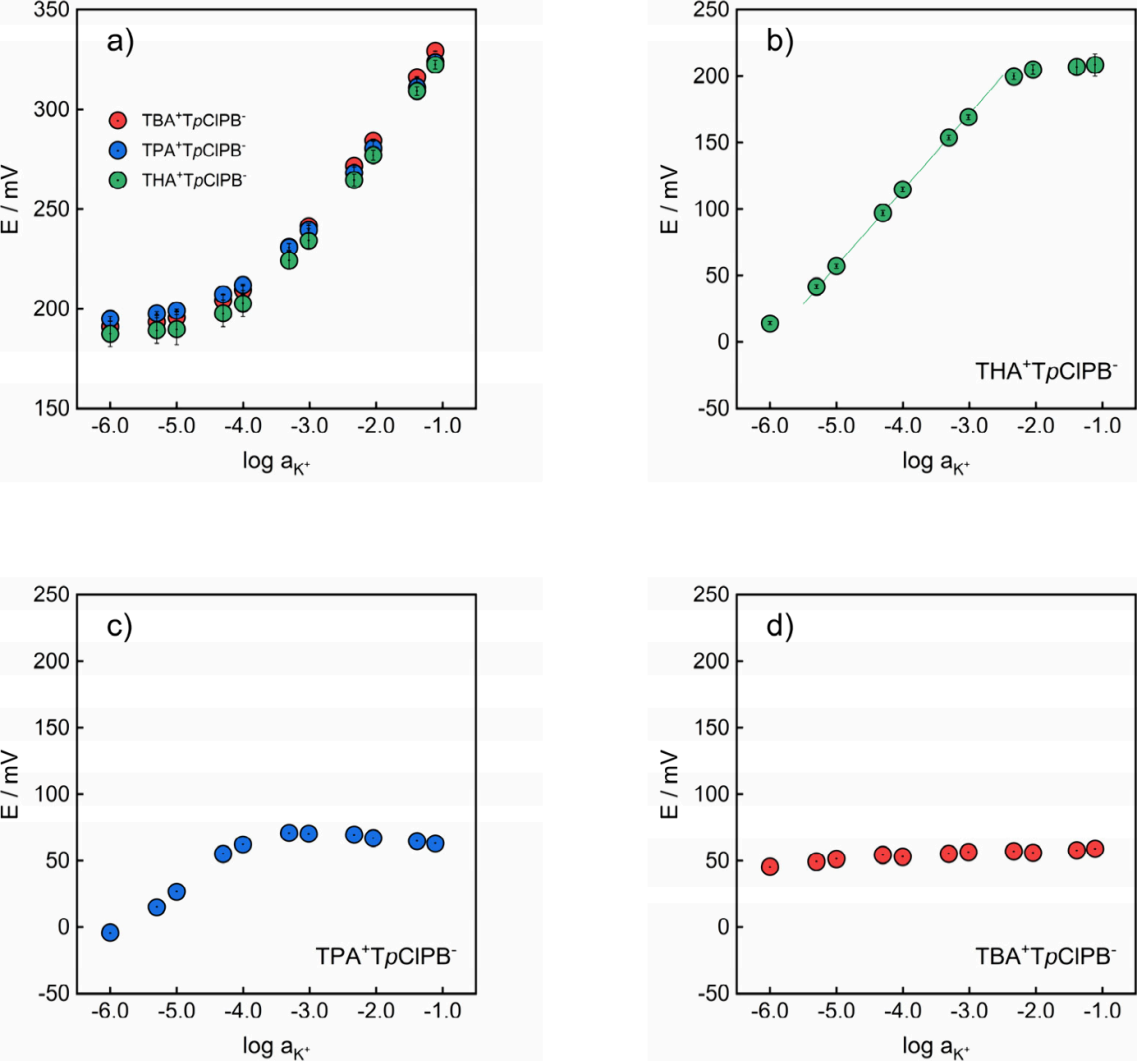
(a) KCl responses of ionophore-free REs based on THAT*p*ClPB, TPAT*p*ClPB and TBAT*p*ClPB (membranes M1–M3, see Table S2). (b–d) KCl responses of REs based on valinomycin and (b) THAT*p*ClPB (membrane M4), (c) TPAT*p*ClPB (membrane M5) and (d) TBAT*p*ClPB (membrane M6). Circle center dots represent the measured EMF; error bars are standard deviations (*n* = 3) acquired across three different electrodes within the same calibration.

As predicted by simulations, the response behavior of membranes without an ionophore was fully governed by the ion mismatch present in the salt (Figure 4a). Moreover, the observed RE responses were practically identical across all electrolyte compositions, confirming the potential-governing role of mismatched salt KT*p*ClPB. The initial potential plateau observed up to 10 μM marks the concentration range for which the distribution of KT*p*ClPB between the two phases remains unaffected by the sample composition. At higher KCl concentrations, the partitioned salt is gradually driven back into the membrane, giving rise to a linear response with sub-Nernstian slopes for all three lipophilic electrolytes: 48.2 mV for TBAT*p*ClPB, 46.5 mV for TPAT*p*ClPB and 48.6 mV for THAT*p*ClPB. The deviation of the observed slopes from theory likely originated from diffusion limitations.

The introduction of valinomycin into the membranes influenced the RE behavior differently, depending on the lipophilicity of the salt. REs based on the most lipophilic THAT*p*ClPB exhibited the least pronounced change with a slight shift of the linear response range to lower KCl concentrations and the appearance of a narrow potential plateau above 5 mM (Figure 4b).

The latter segment indicates the concentration range for which the parallel complexation and ion-exchange processes at the interface eliminate the dependence of the membrane potential on the sample composition. The lower potential plateau limit decreased significantly for REs based on TPAT*p*ClPB (down to 0.1 mM, see Figure 4c), whereas switching to the least lipophilic TBAT*p*ClPB extended the plateau over the entire explored KCl concentration range (Figure 4d). Overall, the observed trend was in satisfying agreement with theoretical predictions, providing experimental confirmation that ionophores indeed could be leveraged to design electrodes with sample independent potentials. The desirable RE potential stability observed during the initial screening prompted a more thorough evaluation of reference membranes incorporating valinomycin with either TPAT*p*ClPB or TBAT*p*ClPB. For this purpose, the lipophilic electrolytes were synthesized with lower ionic mismatches to better reflect the typical purity levels of commercial reagents. A metathesis synthesis was carried out using a modified variation of the previously described potentiometric titration method,^18^ which improved the control over the electrolyte composition (for more details see Supporting Information, Figures S3–S4). TPAT*p*ClPB was synthesized from TPABr and KT*p*ClPB, providing the corresponding salt in quantitative yield with a 1.9 ± 0.6% mismatch (calculated from eq S21). Likewise, TBAT*p*ClPB was obtained from TBACl and KT*p*ClPB with a mismatch of 3.7 ± 2.3%.

Although the ion mismatch was significantly reduced and the byproduct KBr was no longer present in the membrane, the isolated TPAT*p*ClPB did not provide visible improvement compared to that of the initial tests. The RE potential remained relatively stable at KCl concentrations above 0.1 mM; however, with a residual slope of −2.3 ± 0.5 mV and a total RE potential variation of 9.1 mV across three consecutive calibrations of the best performing electrode, the degree of stabilization remained insufficient for realistic applications (Figure S5). In comparison, synthesized TBAT*p*ClPB afforded notably superior RE performance. The corresponding electrode maintained a stable sample-independent potential (slope of 0.4 ± 0.2 mV) between 10 μM and 0.1 M KCl (Figure 5), The response was also highly reproducible between calibrations with a total potential spread of only 2.7 mV across 27 measurements, which is in good agreement with values reported for ionic liquid-based REs (2–4 mV).^14,18–20^ Preliminary testing of RE response in mixed solutions (Figure S6) further demonstrated that, if the K^+^ levels in the sample are sufficient to facilitate the transition of valinomycin-doped membranes to sample-independent response (see Figure 2b), other typical sample ions such as Na^+^ are unlikely to significantly affect the RE function.

**Figure 5:**
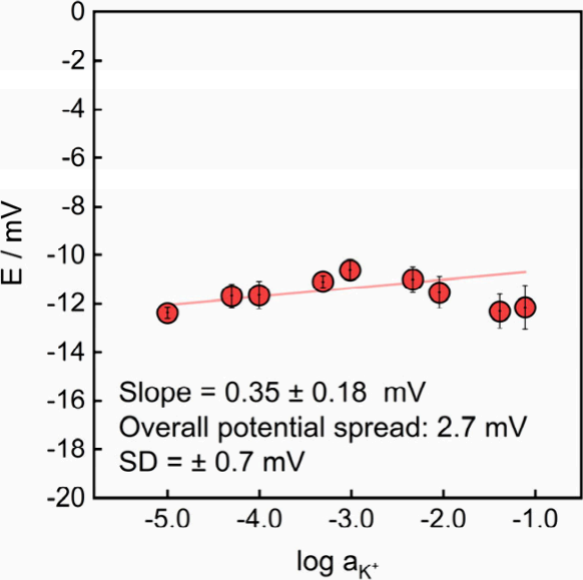
Response of REs based on TBATpClPB and valino­mycin (membrane M8) in KCl solutions. Circle center dots show the measured EMF and error bars are standard deviations (*n* = 3) acquired across three consecutive calibration replicates with the same electrode.

It is worth noting, however, that some variability in stability was observed between smaller reference membranes cut from the same parent membrane (see Figure S7). In addition to that, potential spikes of unclear origin were detected upon stock solution additions throughout RE calibrations (Figure S8). Both phenomena may have a kinetic origin: thickness variation between the membrane segments may influence equilibration during conditioning and measurements, whereas the spikes likely arise from transient perturbation of equilibria at the interface. Literature evidence suggests that diffusion may significantly affect the response of the classical ionic liquid-based REs,^4,21^ and the theoretical treatment published by Egorov et al. earlier this year discusses the magnitude of its influence on RE working limits.^22^ Since diffusion limitations were not considered in the theoretical outline developed in this work, additional theoretical and experimental studies are necessary to further develop the proposed reference electrode principle for real-world applications.

The new reference electrode potential stabilization mechanism introduced here offers a valuable alternative to previously explored principles. The resulting REs are composed of widely available membrane components and do not require elaborate preparation procedures. The high lipophilicity of the membrane components used is expected to mitigate issues related to electrolyte loss and sample contamination. Furthermore, the high selectivity offered by commercially available ionophores is expected to ensure reliable performance across the same range of sample matrices as classical ion-selective electrodes based on similar ligands. These advantages suggest that the proposed approach may represent a promising principle toward the development of robust and practical reference electrodes.

## Supplementary Material



## Notes

Experimental section, RE response simulation protocol and parameters, supplementary simulated curves and other data (PDF)
