# Supplementary material for: Ionophores for Reference Electrodes Based on Organic Electrolytes
Source: Anal Chem. 2025 Nov 7;97(45):24910–5. doi: 10.1021/acs.analchem.5c04958 (PMC12631726; doi:10.1021/acs.analchem.5c04958)
Supplement: Supplementary file 1 [file ac5c04958_si_001.pdf]

Supporting Information for:

Ionophores for Reference Electrodes based on Organic Electrolytes

Nikolai Yu. Tiuftiakov and Eric Bakker*
Department of Inorganic and Analytical Chemistry, University of Geneva, Quai Ernest-Ansermet 30, CH-1211 Geneva, Switzerland

*Corresponding Author: *eric.bakker@unige.ch*

Table of Contents

Reference electrode response simulation **S4**

Membranes containing only mismatched lipophilic electrolyte S4

Membranes incorporating a mismatched lipophilic electrolyte and an ionophore S5

Simulation parameters S6

Figure S1. RE response curves (a), linear (b) and logarithmic (c) membrane concentration profiles simulated over a wider range of sample concentrations for a membrane incorporating a mismatched lipophilic electrolyte P^+R^-. J^+ represents the dominant sample cation. R_T is the total concentration of the lipophilic electrolyte in the membrane, a is the fraction corresponding to the mismatched anion. Square brackets denote membrane concentrations of the corresponding species, c_J is the J^+ concentration in the sample. S7

Figure S2. RE response curves (a), linear (b) and logarithmic (c) membrane concentration profiles and logarithmic sample concentration profiles (d) simulated over a wider range of sample concentrations for a membrane incorporating a mismatched lipophilic electrolyte P^+R^- and an ionophore L selective to a sample cation J^+. The concentration profiles in (b-d) were simulated for the composition corresponding to the blue trace in (a). R_T and L_T are the total membrane concentrations of the lipophilic electrolyte and the ionophore respectively, a is the fraction corresponding to the mismatched anion. Square brackets denote membrane concentrations of the corresponding species, c_i is the sample concentration of ion i. S8

Simplified treatment of the transition from Nernstian response to RE behavior 9

Table S1. Comparison of transition point prediction between the general and the simplified theory. S10

Experimental **S11**

Chemicals S11

Lipophilic electrolyte preparation via potentiometric titrations S11

Figure S3. Experimental setup used for potentiometric titration experiments. S11

Figure S4. Experimental full titration curves (a, c) and the curves corresponding to titrations stopped before the equivalence point (b, d) obtained for TPAT*p*ClPB and TBAT*p*ClPB respectively. Circle center dots represent the measured EMF. The reduced potential range observed with TBAT*p*ClPB is likely caused by the partitioning of the excess TBACl between the organic phase and the aqueous inner filling solutions of WE and RE. S13

Electrode preparation and potentiometric measurements S14

Table S2. Ion-selective membrane compositions used throughout this study. The amounts are relative to a total cocktail mass of 250 mg dissolved in 2 mL THF. S14

Additional data acquired with ionophore-based REs **S15**

Figure S5. The response of REs based on TPAT*p*ClPB and Valinomycin (membrane M7) in KCl solutions. Circle center dots represent the measured EMF, error bars are standard deviations (n = 3) acquired across three consecutive calibration replicates with the same electrode. S15

Figure S7. The KCl response of reference electrodes 2 (a) and 3 (b) prepared using membranes cut out from the same parent membrane as in Fig. 5 (membrane M8). Circle center dots represent the

measured EMF, error bars are standard deviations (n = 3) acquired across three consecutive calibration replicates with the same electrode. S16

Figure S8. Calibration potential-time trace corresponding to one of the calibration replicates from Fig. 5. .... S16

References S17

Reference electrode response simulation

The RE response was simulated for reference membranes incorporating a lipophilic electrolyte P^+R^- with a slight excess of the anionic component, quantified by the mismatch parameter a (the mole fraction of the lipophilic anion not matched by the lipophilic cation). A hydrophilic cation J^+ was assumed to be the predominant cationic species in the aqueous phase, while the influence of its counterion on the interfacial equilibria was considered negligible. For simplicity, activity coefficients in both phases were assumed to be constant and the activities of species were approximated with their respective concentrations. Based on these assumptions, two scenarios were evaluated separately: 1) membranes containing only the mismatched lipophilic salt, and 2) membranes additionally doped with an ionophore selective to J^+.

Membranes containing only mismatched lipophilic electrolyte

To theoretically describe the behavior of reference membranes incorporating a mismatched lipophilic electrolyte, the following system of equations was formulated to represent the equilibria at the sample-membrane interface (Eqs. S1-6):

Partitioning equilibrium of P^+R^- expressed through the corresponding partition constant (Eq. S1):

$$K_{PR} = \frac{[P^+][R^-]}{c_{P^+} c_{R^-}} \tag{S1}$$

where c_i denotes the concentration of the ion i in the aqueous phase, $[i^{z_i}]$ is the concentration of the same ion in the membrane.

Ion-exchange competition between the lipophilic cation P^+ and the sample cation J^+ expressed through their ion-exchange constant (Eq. S2):

$$K_{J/P} = \frac{[J^+] c_{P^+}}{c_{J^+} [P^+]} \tag{S2}$$

Electroneutrality of the polymeric phase (Eq. S3):

$$[P^+] + [J^+] = [R^-] \tag{S3}$$

Mass balance of relevant species and phase distribution (Eqs. S4-6):

$$R_T = [R^-] + q\, c_{R^-} \tag{S4}$$

$$(1 - a)\, R_T = [P^+] + q\, c_{P^+} \tag{S5}$$

$$J_T = c_{J^+} \tag{S6}$$

where R_T represents the total concentration of the lipophilic electrolyte in the membrane, whereas a is the mismatched fraction (anionic excess); J_T is the concentration of the hydrophilic electrolyte dominating the aqueous phase; $q = V_{aq}/V_m$ is the volume ratio of the aqueous and the polymeric phase.

Eqs. S1-6 were solved to determine the concentrations of P^+, R^- and J^+ in both phases as function of the sample composition expressed via J_T. The RE response curves were obtained by inserting the resulting expressions into the Nernst equation (Eq. S7):

$$E - E_i^0 = s \log \frac{c_i}{[i^{z_i}]} \tag{S7}$$

where s is the established Nernstian slope, z_i is the charge and E_i^0 is the standard potential for the ion *i,* which is a function of the relative free energies of solvation in the sample and the membrane ($E_i^0 = \frac{RT}{z_i F} \ln k_i = -\frac{\mu_i^0(org) - \mu_i^0(aq)}{z_i F}$, where k_i is the single ion partition coefficient of the ion i, $\mu_i^0(org)$ and $\mu_i^0(aq)$ are the chemical standard potentials of the ion i in the respective solvents, and R, T and F have their usual significance).

It is important to note that selecting either J^+, P^+ or R^- as the potential-determining ion yielded response curves of identical shape, differing only by a constant vertical offset, which corresponds to the difference between the standard potentials of the selected ions.

Membranes incorporating a mismatched lipophilic electrolyte and an ionophore

Upon incorporation of an ionophore (L) into the reference membrane, Eqs. S1-2, S5-6 remain valid. However, the system must be updated to account for the ion-ionophore complexation along with the associated mass balance of the ionophore within the organic phase. These are introduced as follows (Eqs. S8-10):

Complexation equilibrium of J^+ by L expressed via the complex stability constant (Eq. S8):

$$\beta = \frac{[JL^+]}{[J^+][L]} \tag{S8}$$

Revised electroneutrality condition (Eq. S9):

$$[P^+] + [J^+] + [JL^+] = [R^-] \tag{S9}$$

Ionophore mass balance in the membrane (Eq. S10):

$$L_T = [JL^+] + [L] \tag{S10}$$

where L_T denotes the total ionophore concentration in the membrane.

Additionally, it is assumed that the highly lipophilic ionophore would significantly suppresses the partitioning of lipophilic anion R- into the aqueous phase. Therefore, the respective membrane concentration could be approximated with the total lipophilic electrolyte concentration. This is reflected in the substitution of Eq. S4 with its simplified form (Eq. S11):

$$R_T = [R^-] \tag{S11}$$

The expanded system (Eqs. S1-2,5-6,8-11) was solved to obtain concentrations of P^+, R^- and J^+ in both phases, as well as the complexed (JL^+) and free (L) fractions of the ionophore in the membrane, as function of the sample composition expressed via J_T. As previously, the RE response curves were obtained by inserting the resulting expressions into the Nernst equation (Eq. S7), producing identically shaped curves for different potential-governing ions.

Simulation parameters

The following parameters were used to calculate the electrode response curves from the acquired analytical solutions:

$R_T = 50\ mmol\ kg^{-1}$
$a = 0.1$ (10% mismatch)
$q = 10^4$
$L_T = 10\ mmol\ kg^{-1}$
$\log \beta = 10^{10}$ [4]
$\log K_{J/P} = -9, -11, -13$ ($\log K_{TBA/K} = -9.16$ from modified separate solution method [5])

The organic salt partition constant was estimated for different electrolytes by using the partition constant of J^+R^- ($\log K_{KTpClPB} = 6$ [6]) and the ion-exchange constant according to Eq. S12:

$$\log K_{PR} = \log K_{JR} - \log K_{J/P} \qquad \text{(S12)}$$

$\log K_{PR} = 15, 17, 19$

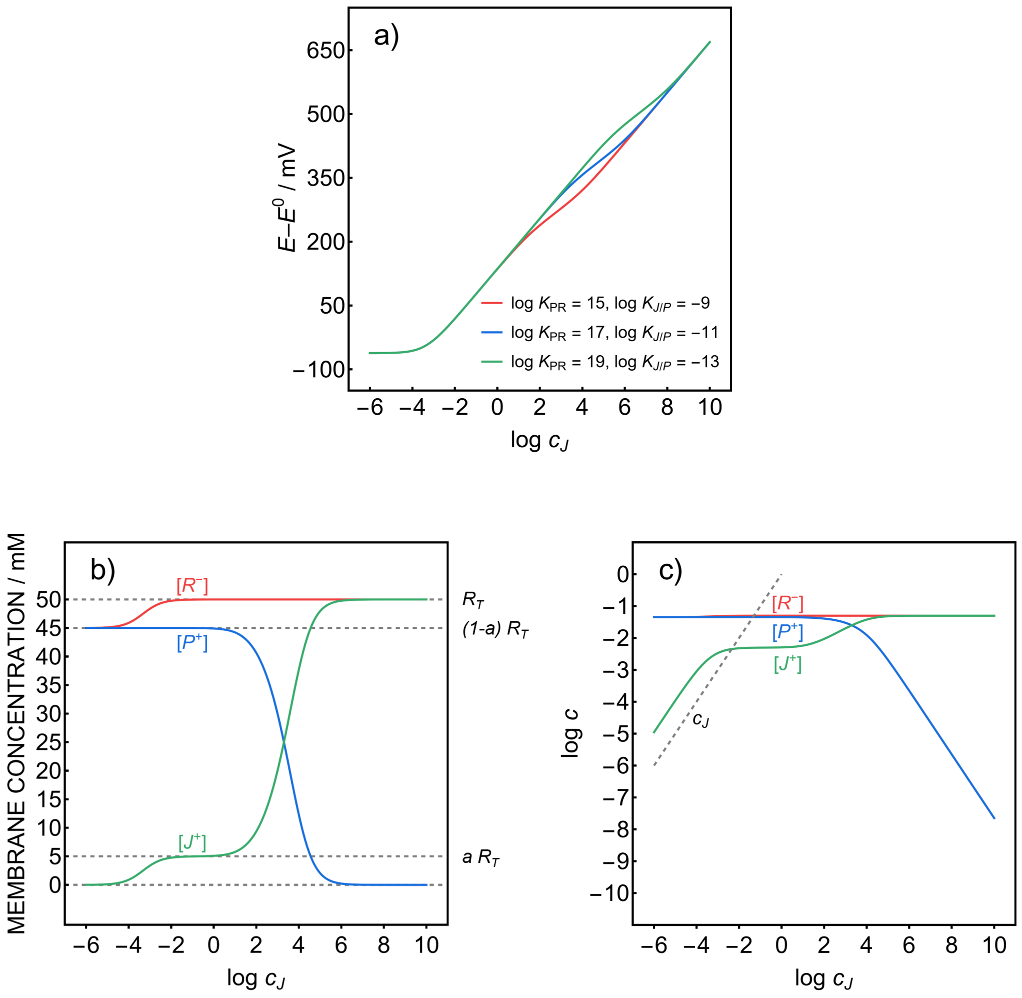

Figure S1. RE response curves (a), linear (b) and logarithmic (c) membrane concentration profiles simulated over a wider range of sample concentrations for a membrane incorporating a mismatched lipophilic electrolyte P^+R^-. J^+ represents the dominant sample cation. R_T is the total concentration of the lipophilic electrolyte in the membrane, a is the fraction corresponding to the mismatched anion. Square brackets denote membrane concentrations of the corresponding species, c_J is the J^+ concentration in the sample.

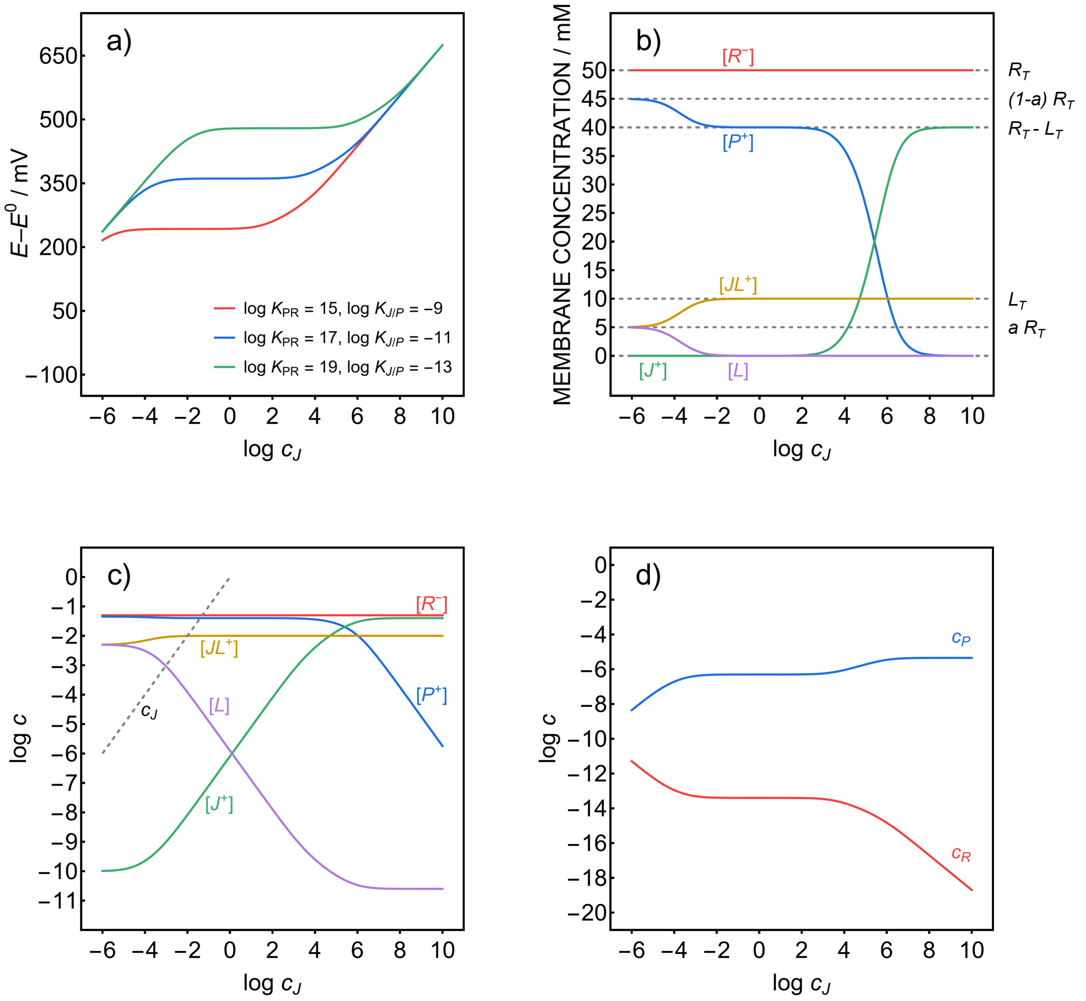

Figure S2. RE response curves (a), linear (b) and logarithmic (c) membrane concentration profiles and logarithmic sample concentration profiles (d) simulated over a wider range of sample concentrations for a membrane incorporating a mismatched lipophilic electrolyte P^+R^- and an ionophore L selective to a sample cation J^+. The concentration profiles in (b-d) were simulated for the composition corresponding to the blue trace in (a). R_T and L_T are the total membrane concentrations of the lipophilic electrolyte and the ionophore respectively, a is the fraction corresponding to the mismatched anion. Square brackets denote membrane concentrations of the corresponding species, c_i is the sample concentration of ion i.

Simplified treatment of the transition from Nernstian response to RE behavior

The simplified theoretical description of the transition point from Nernstian to reference electrode behavior with ionophore-based REs is based on the previously proposed treatment of ISE lower detection limit and stability breakdown of REs based on moderately lipophilic electrolytes [7–9]. The approach considers the Nernstian and reference electrode regions separately and determines the transition point as the intersection of the corresponding response curves.

From the general simulation results, the concentration of the organic cation P^+ remaining in the membrane in the reference electrode region is expressed as follows:

$$[P^+] = R_T - L_T \tag{S13}$$

Eq. S13 may be inserted into Eq. S5 to formulate the P^+ mass balance condition for the corresponding region:

$$(1 - a)\, R_T = R_T - L_T + \; q\, c_{P^+} \tag{S14}$$

Eq. S14 can now be solved to yield the following expression for the aqueous concentration of P^+ in the reference regions:

$$c_{P^+} = q(\, L_T - a\, R_T) \tag{S15}$$

The phase boundary potential in the reference electrode region (Eq. S16) can then be obtained by inserting Eq. S13 and Eq. S15 into the Nernst equation (Eq. S7):

$$E = E^0_{P^+} + s \log \frac{q(\, L_T - a\, R_T)}{R_T - L_T} \tag{S16}$$

We may now consider the Nernstian response region of the same RE. In this case, the concentrations of P^+ in both phases are dictated simultaneously by ion-exchange with J^+ as well as the J^+ complexation equilibrium, which can be described by combining the two corresponding constants (Eq. S2 and S8) into one general expression:

$$K_{J/P}\, \beta = \frac{[JL^+] c_{P^+}}{c_{J^+}[L][P^+]} \tag{S17}$$

Eq. S7 and Eq. S17 can be simplified to obtain an expression for the phase boundary potential as a function of J^+ partitioning:

$$E = E^0_{P^+} + s \log \left(K_{J/P}\, \beta \frac{c_{J^+}[L]}{[JL^+]} \right) \tag{S18}$$

In analogy to classical ISEs, the extraction of J^+ into the membrane and its complexation into JL^+ in the Nernstian region is governed by the available anionic sites, which originate from the anionic mismatch in the lipophilic electrolyte: $[JL^+] = a\, R_T$, $[L] = L_T - a\, R_T$. This allows one to simplify Eq. S18 to Eq. S19:

$$E = E^0_{P^+} + s \log \left(K_{J/P}\, \beta \frac{c_{J^+}(L_T - a\, R_T)}{a\, R_T} \right) \tag{S19}$$

Following the definition of the lower detection limit of ISEs, the transition point between the two electrode functions is found by setting the potential expressions describing the Nernstian response (Eq. S19) and the reference electrode response (Eq. S16) equal. This yields the intersection of the two linear segments of the general electrode response function, corresponding to the lower J^+ concentration limit of the reference electrode region (also presented as Eq. 1 in the main text):

$$c_{J^+}(transition) = \frac{a\, R_T}{K_{J/P}\, \beta\, q\, (R_T - L_T)} \tag{S20}$$

Table S1. Comparison of transition point prediction between the general and the simplified theory.

log K_{PR} (P^+R^- lipophilicity)	log c_J (transition)	
	General theory	Eq. S20
15 (red trace in Fig. 2b)	-5.90	-5.90
17 (blue trace in Fig. 2b)	-3.90	-3.90
19 (green trace in Fig. 2b)	-1.90	-1.90

Experimental

Chemicals

The chemicals used for membrane preparation, namely poly(vinyl chloride) (PVC), 2-nitrophenyl dodecyl ether (NPDE) and tetrahydrofuran (THF) were of Selectophore grade (Sigma-Aldrich). Membrane components potassium tetrakis(4-chlorophenyl)borate (KT*p*ClPB, Selectophore), tetrabutylammonium chloride (TBACl, 97%), tetrapentylammonium bromide (TPABr, 99%), tetrahexylammonium bromide (THABr, 99%) and valinomycin (Selectophore) were also sourced from Sigma-Aldrich. Solvents for potentiometric titrations 1,2-dichloroethane (DCE, 99.8%) and ethyl acetate (EtOAc, 99%) were sourced from Sigma-Aldrich and Fisher Chemical respectively. Dichloromethane (DCM) used in lipophilic salt workup was also obtained from Fisher Chemical. Potassium chloride (KCl) and potassium bromide (KBr) were purchased from Sigma-Aldrich. All salts were of analytical grade or higher. All aqueous solutions were prepared with deionized water (> 18.2 MΩ·cm).

Lipophilic electrolyte preparation via potentiometric titrations

Lipophilic electrolytes with reduced ionic mismatch were synthesized using potentiometric titrations to improve the precision of fixing the cation-to-anion stoichiometry. The procedure followed a previously established method [1–3]. In this approach, an organic solution of a lipophilic electrolyte is placed between two aqueous solutions with different electrolyte activities, separated from the organic phase with porous membranes, and the potential is monitored. Titrating any excess of lipophilic ions in the organic phase with an ion-exchanger of opposite charge results in a switch between cation and anion permselectivity of the liquid membrane at the equivalence point, giving rise to a sharp potential change.

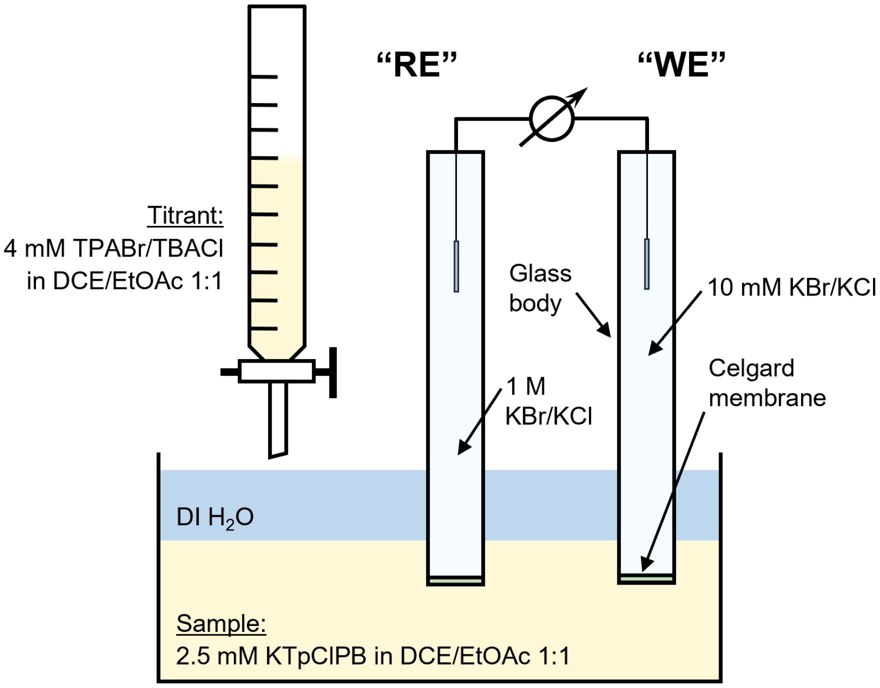

Figure S3. Experimental setup used for potentiometric titration experiments.

The setup used in the present study is shown in Fig. S3. Potentiometric titrations were conducted in a stirred solution at room temperature using a high impedance 16 channel EMF monitor (Lawson Laboratories, Malvern, PA) under a fume hood. Ag/AgCl wires were used as "indicator" and "reference" electrodes. Both electrodes were inserted into glass bodies sealed on the opposite end with microporous polypropylene membranes Celgard 2500 (Celgard, Charlotte, NC) which were held in place by rubber o-rings. The use of such a porous diaphragm allowed to establish an interface between the organic "sample" solution and the aqueous electrode filling solutions while simultaneously preventing the loss of internal fillings and the formation of aqueous droplets in the organic phase. The electrodes were filled with the following aqueous inner filling solutions for the synthesis of the corresponding lipophilic salts: 1 M KBr for the "reference" and 10 mM KBr for the "indicator" electrode with 1 mM KCl as background electrolyte in both (TPAT*p*ClPB); 1 M KCl for the "reference" and 10 mM KCl for the "indicator" electrode (TBAT*p*ClPB). 2.5 mM KT*p*ClPB in DCE/EtOAc 1:1 was used as the sample and titrated with 4 mM TPABr/TBACl dissolved in the same solvent mixture. The organic KT*p*ClPB solution (20 mL) was topped with 10 mL of deionized water to ensure the removal of the hydrophilic electrolyte (KBr/KCl) from the organic phase. The electrodes were immersed directly into the organic layer.

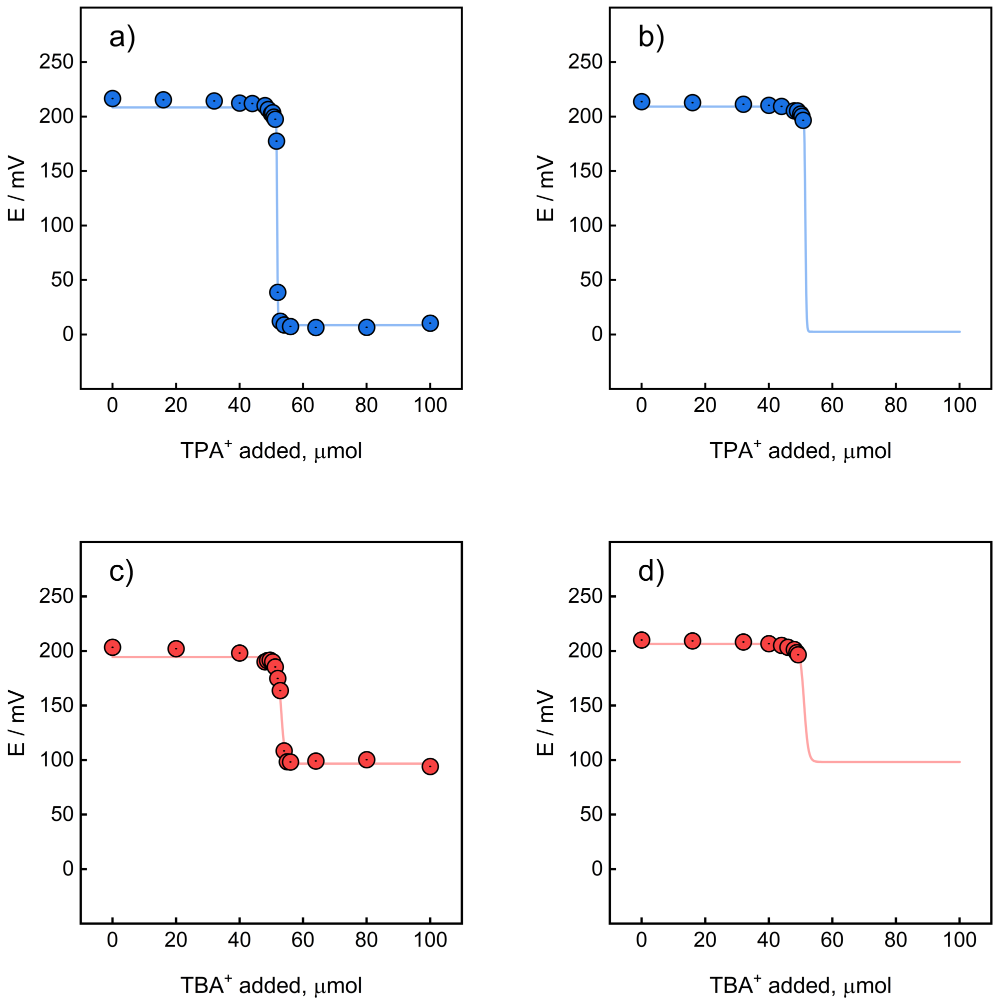

Figure S4. Experimental full titration curves (a, c) and the curves corresponding to titrations stopped before the equivalence point (b, d) obtained for TPAT*p*ClPB and TBAT*p*ClPB respectively. Circle center dots represent the measured EMF. The reduced potential range observed with TBAT*p*ClPB is likely caused by the partitioning of the excess TBACl between the organic phase and the aqueous inner filling solutions of WE and RE.

First, following previously published protocols, full titrations of the anion solution with the corresponding cation solution were performed to precisely identify the equivalence point (Figure S4a,c), which indicated a nearly ideal stoichiometric electrolyte composition. This initial step allowed to account for batch-to-batch variations in the purity of commercial reagents, offering superior control over the electrolyte composition as opposed to preparation via weighing and mixing the components directly. Using the same standardized solutions, separate titrations were performed that were halted at fixed position prior to reaching the equivalence point (Fig. S4b,d). The resulting electrolytes were isolated, which allowed for the preparation of salts with a controlled anionic excess. The molar mismatch parameter of the obtained electrolytes corresponding to the fraction of the anion not compensated by the lipophilic cation (the mismatch parameter) was estimated according to the following expression:

$$a = 100 - \frac{V^{END}}{V^{EP}} \quad \text{(S21)}$$

where V^{END} and V^{EP} are the titrant volumes corresponding to the endpoint of the repeat titrations and the equivalence point obtained from full titrations, respectively.

The titrated mixtures from the latter experiment were joined together and transferred into a separatory flask. The organic and aqueous fractions were separated. The aqueous layer was washed with DCM (3x25 mL). The joint organic fraction was washed with 50 mL brine and dried over a minimal possible amount of $MgSO_4$. The drying agent was then filtered off and the solvent rotary evaporated. Both salts were obtained as white flaky solids in quantitative yields (TBAT*p*ClPB – 175 mg, TPAT*p*ClPB – 113 mg).

Electrode preparation and potentiometric measurements

All membranes were cast by weighing sensing components together with PVC and the plasticizer NPDE (1:1 by weight), to give a total cocktail mass of 250 mg, dissolving the mixture in 2 mL of THF and pouring it into a 2.2-cm-diameter glass ring affixed on a glass slide. The solvent was allowed to evaporate overnight. Three 8 mm disks were cut out of the resulting parent membrane, mounted in commercial electrode bodies (Oesch Sensor Technology, Sargans, Switzerland), filled with the respective inner filling solutions and conditioned overnight in the solutions identical in composition to the internal filling. The membrane compositions and the internal filling solutions used throughout the study are given in Table S2.

Table S2. Ion-selective membrane compositions used throughout this study. The amounts are relative to a total cocktail mass of 250 mg dissolved in 2 mL THF.

Membrane	Membrane components									Inner Filling Solution (KCl)
	PVC[a]	NPDE[a]	KTpClPB[b]	THACl[b]	TPABr[b]	TBABr[b]	TPAT*p*ClPB[b]	TBAT*p*ClPB[b]	Valinomycin[b]	
M1	47.8	47.8	50	45	-	-	-	-	-	1 mM
M2	47.9	47.9	50	-	45	-	-	-	-	
M3	48.1	48.1	50	-	-	45	-	-	-	
M4	47.2	47.2	50	45	-	-	-	-	10	
M5	47.4	47.4	50	-	45	-	-	-	10	
M6	47.6	47.6	50	-	-	45	-	-	10	
M7	47.8	47.8	-	-	-	-	50	-	5	10 mM
M8	48.0	48.0	-	-	-	-	-	50	5	

[a] Mass percentage; [b] mmol kg^{-1}

All potentiometric calibrations were performed with a high impedance 16 channel EMF monitor (Lawson Laboratories, Malvern, PA) using a double-junction Ag/AgCl/3M KCl/1M LiOAc reference electrode (Metrohm, Switzerland).

For calibrations in KCl solutions, the conditioned electrodes were immersed in the most dilute solution and upon complete potential equilibration (5-10 min), aliquots of the analyte standard solution were added to the analyzed solution. Calibrations with the same electrodes were performed once (averaging between 3 electrodes) during the initial lipophilic electrolyte screening and repeated 3 times (averaging between different calibrations for the same electrode) throughout further tests.

Additional data acquired with ionophore-based REs

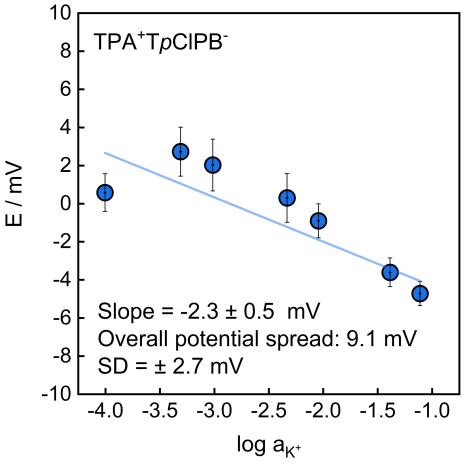

Figure S5. The response of REs based on TPAT*p*ClPB and Valinomycin (membrane M7) in KCl solutions. Circle center dots represent the measured EMF, error bars are standard deviations (n = 3) acquired across three consecutive calibration replicates with the same electrode.

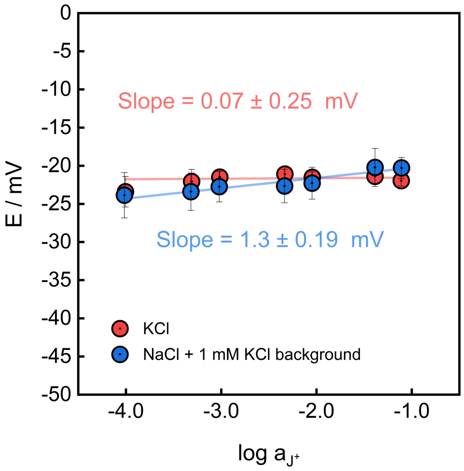

Figure S6. The response of REs based on TBAT*p*ClPB and Valinomycin (membrane M8) in pure KCl solutions (red) and NaCl solutions with a constant 1 mM KCl background (blue). Circle center dots represent the measured EMF; error bars are standard deviations (n = 3) acquired across three consecutive calibration replicates with the same electrode.

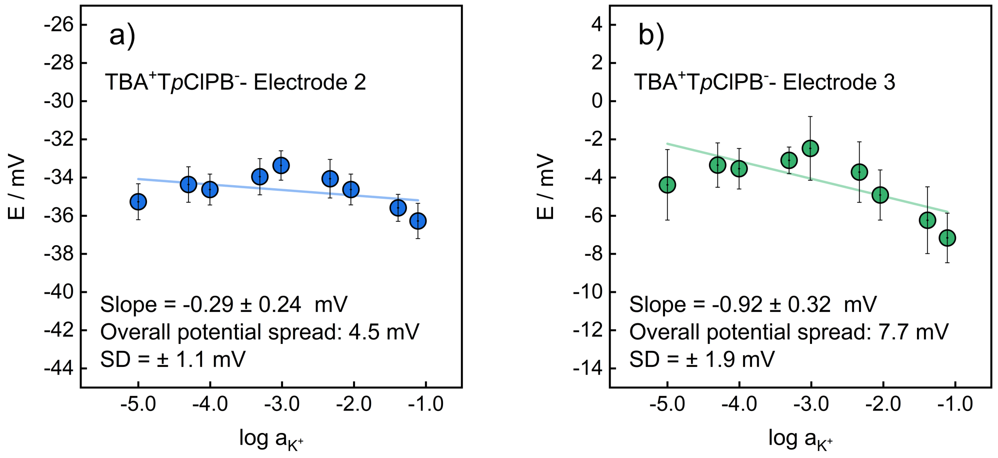

Figure S7. The KCl response of reference electrodes 2 (a) and 3 (b) prepared using membranes cut out from the same parent membrane as in Fig. 5 (membrane M8). Circle center dots represent the measured EMF, error bars are standard deviations (n = 3) acquired across three consecutive calibration replicates with the same electrode.

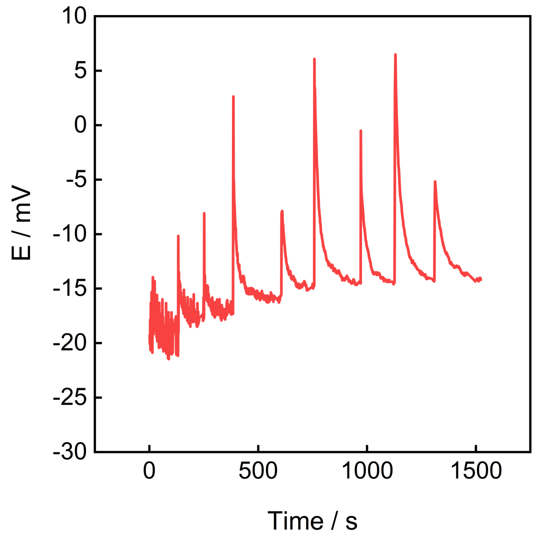

Figure S8. Calibration potential-time trace corresponding to one of the calibration replicates from Fig. 5.

References

(1) Silvester, D. S.; Grygolowicz-Pawlak, E.; Bakker, E. Assessing Ion-Exchange Properties and Purity of Lipophilic Electrolytes by Potentiometry and Spectrophotometry. *Electrochem. Commun.* **2010**, *12* (1), 110–113. https://doi.org/10.1016/j.elecom.2009.10.048.

(2) Silvester, D. S.; Grygolowicz-Pawlak, E.; Bakker, E. Potentiometric Determination of Coextraction Constants of Potassium Salts in Ion-Selective Electrodes Utilizing a Nitrobenzene Liquid Membrane Phase. *Anal. Chim. Acta* **2010**, *683* (1), 92–95. https://doi.org/10.1016/j.aca.2010.10.012.

(3) Tiuftiakov, N. Yu.; Bakker, E. Critical Influence of Organic Salt Purity on the Performance of Reference Electrodes Based on Highly Lipophilic Electrolytes. *Sens. Actuators B Chem.* **2025**, *438*, 137795. https://doi.org/10.1016/j.snb.2025.137795.

(4) Qin, Y.; Mi, Y.; Bakker, E. Determination of Complex Formation Constants of 18 Neutral Alkali and Alkaline Earth Metal Ionophores in Poly(Vinyl Chloride) Sensing Membranes Plasticized with Bis(2-Ethylhexyl)Sebacate and o-Nitrophenyloctylether. *Anal. Chim. Acta* **2000**, *421* (2), 207–220. https://doi.org/10.1016/S0003-2670(00)01038-2.

(5) Bakker, E. Determination of Unbiased Selectivity Coefficients of Neutral Carrier-Based Cation-Selective Electrodes. *Anal. Chem.* **1997**, *69* (6), 1061–1069. https://doi.org/10.1021/ac960891m.

(6) Telting-Diaz, M.; Bakker, E. Effect of Lipophilic Ion-Exchanger Leaching on the Detection Limit of Carrier-Based Ion-Selective Electrodes. *Anal. Chem.* **2001**, *73* (22), 5582–5589. https://doi.org/10.1021/ac010526h.

(7) Tiuftiakov, N. Yu.; Zdrachek, E.; Bakker, E. Ion-Exchange and Lipophilicity Limitations of Ionic Liquid Reference Electrodes. *Sens. Actuators B Chem.* **2024**, *407*, 135474. https://doi.org/10.1016/j.snb.2024.135474.

(8) Bakker, E.; Xu, A.; Pretsch, E. Optimum Composition of Neutral Carrier Based pH Electrodes. *Anal. Chim. Acta* **1994**, *295* (3), 253–262. https://doi.org/10.1016/0003-2670(94)80230-0.

(9) Bakker, E. Origin of Anion Response of Solvent Polymeric Membrane Based Silver Ion-Selective Electrodes. *Sens. Actuators B Chem.* **1996**, *35* (1–3), 20–25. https://doi.org/10.1016/S0925-4005(96)02007-2.
